# Outcome of an Accelerated Treatment Algorithm for Patients Developing Diarrhea as a Complication of Ipilimumab-Based Cancer Immunotherapy in a Community Practice

**DOI:** 10.3390/curroncol31060260

**Published:** 2024-06-18

**Authors:** Clarice Ho, Wolfram Samlowski

**Affiliations:** 1 School of Medicine, University of Nevada, Reno, NV 89557, USA; clariceh@med.unr.edu; 2 Comprehensive Cancer Centers of Nevada, Las Vegas, NV 89148, USA; 3 Kerkorian School of Medicine, University of Nevada Las Vegas (UNLV), Las Vegas, NV 89106, USA

**Keywords:** checkpoint inhibitors, diarrhea, inflammatory colitis, infliximab, glucocorticosteroids, ipilimumab, nivolumab

## Abstract

Immune-mediated diarrhea represents a serious complication of checkpoint inhibitor therapy, especially following ipilimumab-based treatment. Efficient diagnosis and control of diarrhea remains an ongoing challenge. We developed an accelerated management paradigm for patients with ipilimumab-induced diarrhea. Patients who developed significant diarrhea (>five loose stools/day) were presumed to be developing immune colitis. Therapy was interrupted and patients were treated with a methylprednisolone dose pack. If diarrhea was not completely resolved, high-dose steroids and infliximab were promptly added. Only non-responding patients underwent further evaluation for infection or other causes of diarrhea. A total of 242 patients were treated with ipilimumab-based regimens. Forty-six developed significant diarrhea (19%) and thirty-four (74.4%) had a rapid resolution of diarrhea following glucocorticosteroid and infliximab treatment. The median time to resolution of diarrhea was only 8.5 ± 16.4 days. Accelerated treatment for presumed immune-mediated diarrhea resulted in the rapid control of symptoms in the majority of patients. There were no intestinal complications or deaths. Immunosuppressive therapy for diarrhea did not appear to decrease the remission rate or survival. After the control of diarrhea, most patients were able to continue their planned immunotherapy. Further testing in 11/46 patients with unresponsive diarrhea revealed additional diagnoses, allowing their treatment to be adjusted.

## 1. Introduction

In 2005, the historical median survival of metastatic melanoma patients was 6–7 months following chemotherapy, with only 25% of patients achieving 1-year survival [1,2]. The development of new treatment options, such as immune checkpoint inhibitor (ICI)-directed antibodies, has dramatically improved the treatment outcome in metastatic melanoma. The CTLA 4 (cytotoxic T lymphocyte-associated protein 4) antibody ipilimumab induced a modest objective response rate (10–15%), but it was soon recognized that some patients achieved durable complete remissions [3]. With the 10-year follow-up, approximately 21% of ipilimumab-treated patients remained free of disease [4]. Subsequently, PD-1 (programmed death-1) monoclonal antibodies were developed. These agents, such as nivolumab and pembrolizumab, further increased the remission rate and survival of metastatic melanoma patients [5,6]. The estimated 5-year progression-free survival in previously untreated metastatic melanoma patients with PD-1 monotherapy ranged from 29 to 36%, with a 5-year overall survival of 41–44% [7,8]. Further improvements in outcome resulted from combinations of CTLA-4 plus PD-1 antibodies, such as ipilimumab plus nivolumab. At the 6.5-year follow-up, the Checkmate 067 trial achieved a median survival of 72.1 months [9]. This combination produced a long-term progression-free survival of 34% [9]. More recently, the Alliance EA6134 trial (DreamSeq) demonstrated a significant superiority in progression-free survival for the initial combination ipilimumab/nivolumab therapy versus targeted therapy with dabrafenib plus trametinib, even in BRAF-mutant melanoma [10]. Thus, the combination checkpoint inhibitor therapy has become the de facto standard of care for first-line treatment for metastatic melanoma.

Since ICI agents activate cytotoxic T cells [11], they all have the potential to induce a unique spectrum of immune-related adverse events (irAEs) [12]. These irAEs are typically inflammatory or autoimmune in nature and can affect virtually any organ. Severe and potentially life-threatening toxicities have been reported that involve the skin, lungs, GI system, liver, kidneys, heart, endocrine glands, central nervous system, as well as other organs [12,13]. Combination therapy with nivolumab and ipilimumab did not result in any unique toxicities compared to the treatment with ICI monotherapy, but the frequency of severe (Grade 3–4) irAEs was increased [12,13]. Severe (Grade 3–4) irAEs occurred in about 55% of patients treated with the ipilimumab plus nivolumab combination, versus 27.3% with ipilimumab monotherapy or 16.3% with nivolumab alone [12]. In addition, many patients receiving combination immunotherapy discontinued treatment due to toxicity.

One of the most frequent reasons for treatment discontinuation is the development of severe diarrhea due to bowel inflammation (colitis) [14]. This toxicity is most often seen following the administration of ipilimumab-containing immunotherapy regimens. Diarrhea of any severity occurs in up to 30–40% of patients receiving ipilimumab monotherapy and approximately 45% of patients receiving ipilimumab plus nivolumab treatment [15,16,17,18]. The onset of ICI-induced diarrhea most often occurs within 5–10 weeks after the initiation of ICI therapy (often after the second or third dose). The clinical presentation may include severe watery diarrhea (an increase of >five bowel movements/d), abdominal pain, and cramping. In severe cases, there may be ileus or blood and mucous in the stool [19]. Severe to life-threatening colitis is estimated to occur in 8.4–11% with ipilimumab monotherapy [20] and approximately 11% of patients treated with combination therapy [17]. Severe colitis may produce additional complications such as dehydration, bowel perforations [21,22], hospitalizations (7–24%) [23], or even mortality [24].

Prompt recognition and treatment of diarrhea is paramount to reducing the severity of colitis and the risk of complications. Current management suggestions include laboratory evaluation to exclude infectious etiologies of diarrhea, colonoscopy, and abdominal imaging to help differentiate immune-mediated colitis from other causes of diarrhea [19,25]. Treatment for mild diarrhea with supportive therapies such as hydration and hypomotility agents has been suggested. More severe cases require treatment with immunosuppressive agents such as glucocorticosteroids. For steroid-refractory patients, the addition of tumor necrosis factor (TNF) inhibitors, such as infliximab, is recommended [19,25].

In our community practice, we have observed a low incidence of colonic infections in ICI-treated patients. Due to significant delays in testing for infectious agents and arranging a colonoscopy for evaluation, we developed an accelerated treatment protocol to promptly initiate effective treatment for patients with diarrhea who are developing presumed immune colitis following ICI treatment. The outcomes of this accelerated ICI-induced diarrhea management strategy in metastatic melanoma patients treated with ipilimumab-containing regimens is reported herein. The need for hospitalization and the ability to continue planned ICI therapy after the control of diarrhea was also evaluated in this analysis.

## 2. Materials and Methods

### 2.1. Patient Selection and Data Collection

Records of patients treated by a single physician (WS) between 3/2012 and 3/2022 were screened. These records were contained in a secure Health Information Portability and Accessibility Act (HIPAA)-compliant iKnowMed database (McKesson, Houston, TX, USA), which was searched for patients who had received treatment with infliximab or vedolizumab. Records were individually reviewed to identify patients who had received ipilimumab-containing regimens as treatment for malignancy and had developed diarrhea. A presumptive diagnosis of immune-mediated diarrhea was based on clinical symptoms (new onset or a marked increase in the frequency of watery loose stools >5/day compared to pretreatment). Since most patients did not undergo colonoscopy to formally diagnose colitis, the term diarrhea is used to describe this toxicity. Patients were excluded from the analysis if they received infliximab or vedolizumab for conditions other than diarrhea associated with ICI administration (e.g., arthritis), did not have a cancer diagnosis, or did not receive an ipilimumab-containing ICI regimen.

### 2.2. Clinical Characteristics and Outcomes of Patients

Data from the individual records of patients were extracted into a spreadsheet (Excel, Microsoft, Redmond, WA, USA, version 16.83) for analysis. A unique patient number was assigned to each patient. Demographic characteristics of patients extracted from electronic medical records included age, gender, and comorbid medical conditions. Oncologic variables, such as cancer stage, primary and metastatic sites, the ICI agent, the treatment start date, the treatment end date, and the total number of ICI doses administered, were recorded. The date of progression, date of death (if applicable), and any subsequent treatments were also extracted from the computer record. ICI toxicity was recorded. This included the date of onset of diarrhea and the timing of diarrhea onset related to the ipilimumab treatment. Treatment with infliximab or vedolizumab, including dosage and duration, time to resolution of diarrhea, and hospitalization data, was recorded. The number of doses of infliximab or vedolizumab needed to control diarrhea was noted. In patients whose diarrhea did not respond to infliximab, other causes for diarrhea identified by subsequent testing were also recorded. Whether planned ICI treatment was able to be resumed after the resolution of diarrhea was also documented. The data spreadsheet was deidentified following data extraction. A review of the study design was performed by the Western institutional review board (IRB) chair. This retrospective data review was deemed exempt from full IRB review.

Clinical responses were analyzed from the start of the ipilimumab-containing regimen that triggered the diarrhea. Responses were graded based on the RECIST 1.1 criteria [26]. A complete response (CR) was characterized by the disappearance of all target and non-target lesions and the normalization of tumor marker levels. A partial response (PR) was characterized by a more than 30% decrease in the sum of bidimensional tumor dimensions on radiographic imaging. Progressive disease (PD) was characterized by a greater than 20% increase in the sum of bidimensional tumor measurements or the development of new metastatic sites. Stable disease (SD) was characterized as a response that did not meet the criteria for CR, PR, or PD. Adverse events were graded using the National Cancer Institute Common Toxicity Criteria for Adverse Events (CTCAE 5.0) criteria [27].

### 2.3. Treatment Regimens

Cancer immunotherapy treatment regimens evolved over the duration of this study. Patients were treated with a variety of different intravenous ipilimumab regimens employing doses of 3 mg/kg every 3 weeks, a fixed dose 240 mg every 2 weeks, or a fixed dose of 480 mg every 4 weeks. Some of our patients were treated with combined therapy using either the standard (3 mg/kg ipilimumab plus 1 mg/kg nivolumab i.v. every 3 weeks) or an alternate regimen of ipilimumab (1 mg/kg ipilimumab plus 3 mg/kg nivolumab i.v. every 3 weeks) for 4 doses with subsequent nivolumab maintenance [28].

If patients progressed on ICI therapy and had targetable mutations (BRAF, NRAS, and NF1), these patients were offered the addition of a low-dose targeted therapy (TT) with the continuation of PD-1 antibody therapy. This included addition of a TT of dabrafenib 75 mg/day, encorafenib 75 mg/day, trametinib 1 mg/day, or binimetinib 15 mg b.i.d. Cautious dose escalation of BRAF or MEK inhibitors was considered if not toxicity was observed [29,30].

### 2.4. Accelerated Diarrhea Treatment Schema

**Figure 1:**
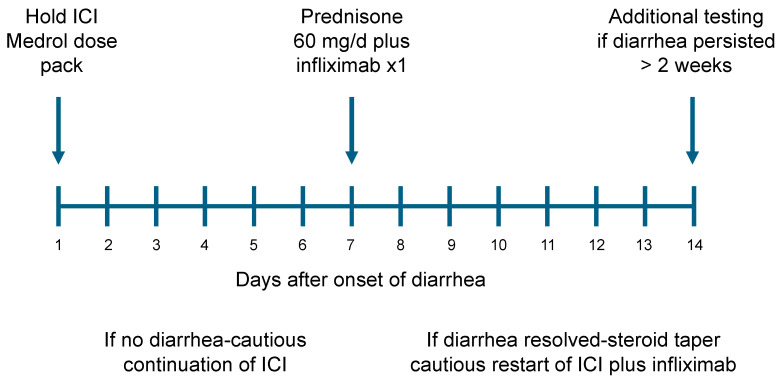
Checkpoint inhibitor-induced diarrhea treatment schema.

When a patient receiving ICI therapy reported the new onset of diarrhea, immunotherapy was interrupted. Patients with diarrhea were then prescribed methylprednisolone on a 6-day taper (Medrol dose pack) (Figure 1: treatment schema). The initial dose was 24 mg of methylprednisolone per day (six 4 mg tablets), with a daily taper of 4 mg/d. If the diarrhea resolved and did not recur after a rapid steroid taper, patients were thought unlikely to have immune colitis and continued the planned ICI treatment. If diarrhea responded only transiently, patients were started on high-dose steroids (prednisone 60 mg/d). The next step in our treatment algorithm was to add infliximab (5 mg/kg iv) within 1–2 weeks (due to the need for insurance approval). If diarrhea subsided after infliximab administration, a slow steroid taper was begun (10 mg/week). Once patients reached 20 mg or less of prednisone/day without diarrhea, the resumption of the planned ICI therapy was considered (usually with continuing infliximab treatment). In the infrequent patients whose diarrhea did not improve or resolve with this management after 2 weeks, further diagnostic evaluation was then pursued, including stool testing for clostridium difficile, other bacterial stool pathogens, and colonoscopy. ICI treatment was paused in these patients and diarrhea treatment was subsequently modified to encompass any additional pathology identified.

### 2.5. Statistical Analysis

Descriptive statistics were calculated using an Excel spreadsheet (Version 16.83, Microsoft, Redmond, WA, USA) and expressed as a data range, median, and standard deviation. Progression-free and overall survival were evaluated using the methods described by Kaplan and Meier [31]. A log-rank test was used to compare the survival curves [32]. The data analysis cutoff date was 3 January 2022.

## 3. Results

### 3.1. Patient Characteristics

**Figure 2:**
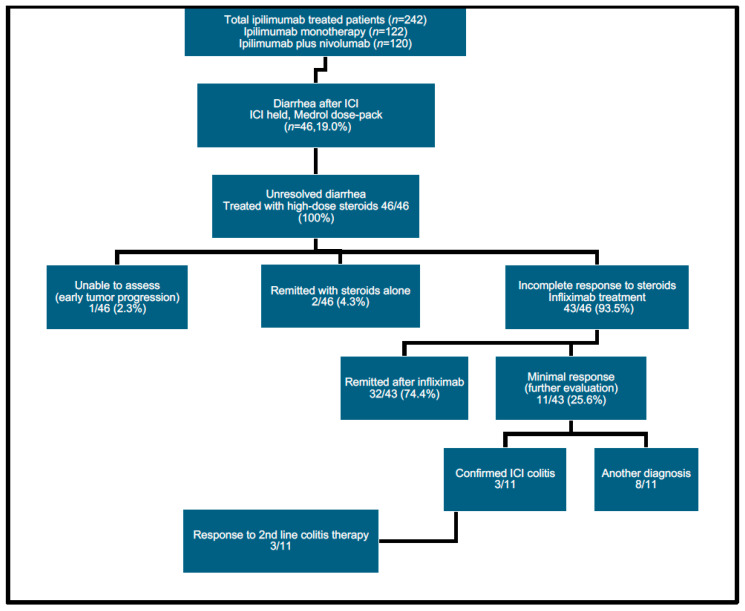
CONSORT diagram of patient evaluation.

Between 3/2012 and 3/2022, 242 patients were treated at this institution with ipilimumab-containing regimens (122 with ipilimumab monotherapy and 120 with ipilimumab plus nivolumab). Our retrospective chart review identified 46/242 patients (19%) who developed clinically significant diarrhea during treatment. These 46 patients form the basis for the current analysis. All patients were managed using an accelerated treatment algorithm (Figure 2: CONSORT flowchart). Individual characteristics of patients who developed diarrhea are shown (Table 1). Their median age at the start of the ICI therapy was 62 years old, with an age range of 25 to 85 years old. A total of 61% of the patients were male and 39% were female. A total of 43 patients were treated for melanoma, 2 were treated for renal cell carcinoma, and 1 was treated for prostate cancer.

Of the 46 patients who developed clinically significant diarrhea, 22 had received treatment with ipilimumab monotherapy and 20 with ipilimumab plus nivolumab regimens. Another four patients were eventually retreated after disease progression with a second cycle of ipilimumab-containing immunotherapy regimens prior to the onset of diarrhea. Of the latter four patients, diarrhea developed after receiving ipilimumab followed by ipilimumab plus nivolumab (*n* = 1), retreatment with ipilimumab monotherapy (*n* = 2), or a second cycle of ipilimumab plus nivolumab (*n* = 1).

### 3.2. Diarrhea Onset and Treatment

**Figure 3:**
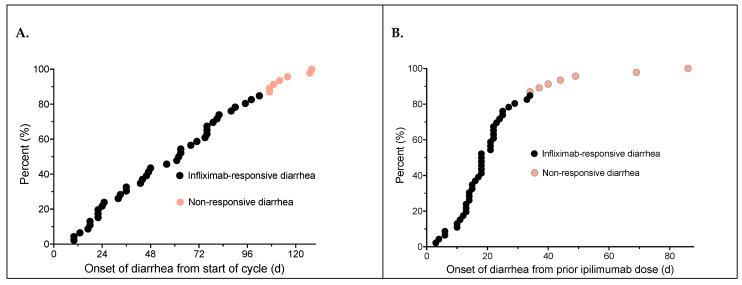
Time to diarrhea onset from the initial ipilimumab dose of the treatment cycle (**A**); time to diarrhea onset from the ipilimumab dose that triggered diarrhea (**B**). Patients who had refractory diarrhea and underwent further workup identifying other causes of diarrhea are shown in orange.

The number of ICI doses patients received prior to diarrhea onset and the time to diarrhea onset from both the start of treatment and from the preceding ipilimumab dose are shown in Table 1. The median time to onset of diarrhea from the start of their ipilimumab-based regimen was 62.5 ± 34.5 days (range 10–128 days) (Figure 3A). The median time to onset of diarrhea from the triggering ipilimumab dose was only 18.0 ± 7.2 days (range 3–69 days) (Figure 3B). It should be noted that patients who had non-responsive diarrhea to steroids and infliximab generally had a more delayed onset of diarrhea from both from the ICI regimen start date as well as having onset >30 days following the last preceding ipilimumab dose.

**Figure 4:**
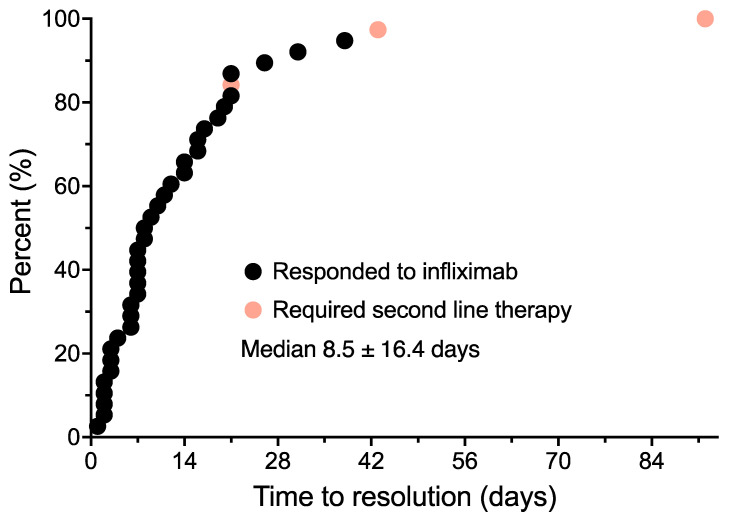
Time to resolution of diarrhea from the initial infliximab dose. Patients who had refractory diarrhea and were found to have checkpoint inhibitor-induced diarrhea are shown in orange.

Patients whose diarrhea failed to respond to a methylprednisolone dose pack were escalated to treatment with high-dose oral prednisone. Two patients were remitted with high-dose prednisone alone before they could receive infliximab. These patients subsequently received infliximab with subsequent cycles of immunotherapy to prevent the recurrence of diarrhea, and are therefore included in the analysis. One additional patient was unable to be assessed due to early progression and death due to melanoma. Another 43 patients responded transiently to steroids and were then treated with infliximab within 1–2 weeks. The individual treatment outcome for each patient is shown in Table 2. The number of infliximab doses received, the time to resolution of diarrhea in days, and immunotherapy doses received post-treatment are shown. The median time from the onset of diarrhea to infliximab treatment initiation was 7.5 ± 19.4 days. Of the 43 patients treated with infliximab, 33 patients (72.7%) had a rapid remission of their diarrhea. The median time to resolution of diarrhea from the infliximab dose was 8.5 ± 16.4 days (Figure 4). This included three patients who required a second infliximab dose to fully control their diarrhea following significant improvement after the initial infliximab dose. A total of 10 patients treated with infliximab had no response and underwent further diagnostic evaluation. Three of the ten patients were confirmed to have immune colitis by colonoscopy. All three patients subsequently responded to the vedolizumab treatment. Seven patients had additional causes for diarrhea identified. These included ischemic colitis (*n* = 1), clostridium difficile colitis (*n* = 3), lymphocytic colitis (*n* = 1), and diarrhea from dabrafenib/trametinib treatment (*n* = 1), and unresponsive diarrhea from an unknown etiology (*n* = 1).

Once the diarrhea was controlled, 30/43 patients (70%) received additional planned doses of immunotherapy treatment (median of 4.5 ± 6.2 doses). Five patients were not planned to receive additional immunotherapy treatment post-diarrhea, as they had already completed a four-dose ipilimumab monotherapy regimen. The other eight patients did not continue the immunotherapy due to tumor progression.

### 3.3. Outcomes and Analysis of Overall Survival

Of the patient cohort, 11 patients were hospitalized following the ICI treatment. Seven of these hospitalizations were related to diarrhea. The reasons for diarrhea-related hospitalization in seven patients included insurance denial of outpatient infliximab treatment (*n* = 3), the delayed initiation of treatment due to patient factors (*n* = 1), atypical presentations of colitis (*n* = 2), and the management for diarrhea and concurrent hypopituitarism (*n* = 1). Four patients were hospitalized due to other conditions not related to diarrhea (Table 2). There were no complications or deaths due to diarrhea in our patient series.

**Figure 5:**
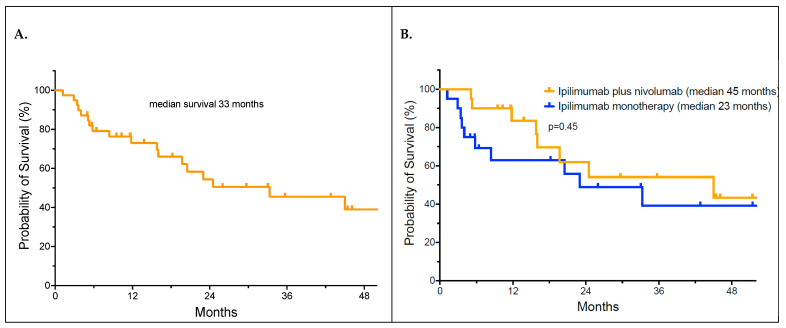
Overall survival of patients who developed checkpoint inhibitor-induced diarrhea (**Panel A**); overall survival of patients by ipilimumab monotherapy versus combination therapy (**Panel B**).

We evaluated whether immunosuppression with steroids and infliximab affected survival. The median overall survival of patients with infliximab-responsive diarrhea was 33 months by the Kaplan–Meier analysis (Figure 5A). The median overall survival for those who received ipilimumab monotherapy was 23 months versus 45 months for those receiving a combination of ipilimumab plus nivolumab (Figure 5B). The difference in the median survival between the two therapy groups did not reach statistical significance (*p* = 0.45 by the log-rank test). After 24 months, there were no further relapses of melanoma in responding patients.

## 4. Discussion

Currently, the National Comprehensive Cancer Network’s (NCCN) guidelines for the management of immunotherapy-related toxicities (version 1.2024) recommend testing stool for C. difficile, ova and parasites, and other bacteria and viruses when patients develop a significant increase in diarrhea following immunotherapy [33]. For mild diarrhea (Grade 1), it is suggested that therapy should be interrupted, and the administration of loperamide or diphenoxylate/atropine is recommended [33]. In instances of moderate diarrhea (Grade 2), additional assessment suggestions include possible abdominal and pelvic CT scans (with contrast), and gastrointestinal consultation for endoscopy. Steroid treatment (1–2 mg/kg methylprednisolone or prednisone) is recommended, followed by a slow taper. In Grade >2 diarrhea, it is also advised to test stool for inflammatory markers, such as lactoferrin and calprotectin [34]. Further treatment recommendations include the utilization of budesonide if there is confirmation of microscopic colitis or, alternatively, oral high-dose steroid administration. Monitoring of diarrhea is suggested for 3 days, and if there is no improvement, escalation to IV steroids, and then the addition of infliximab or vedolizumab is recommended [33,34]. For the management of very severe diarrhea (Grade 3–4), immediate initiation of IV methylprednisolone is recommended. If there is minimal response in 1–2 days, transition to oral steroids and additional immunosuppression with the addition of infliximab and vedolizumab may be necessary [33,34].

We have found that, in our patient population, the risk of identifiable enteric infection has been low. Due to difficulties obtaining timely stool testing results and arranging colonoscopy in a community practice setting, we developed a simplified and accelerated treatment approach for significant ICI-induced diarrhea. One cannot overemphasize the need for an effective toxicity reporting mechanism to be utilized by patients. Upon recognition of diarrhea (increase >five loose or watery stools/day), patients were immediately started on a methylprednisolone dose pack with monitoring over the next 6 days during the steroid taper. The use of hypomotility agents was avoided, as they tended to confuse the evaluation of clinical responses to the steroid treatment. If diarrhea resolved completely following the methylprednisolone dose pack administration and did not recur, this was felt to be unlikely to represent a treatment side-effect. In these patients, ICI treatment was cautiously resumed.

If patients had an incomplete response of diarrhea to the Medrol dose pack, high-dose steroids (60 mg/d of prednisone) were started, and infliximab treatment was arranged as soon as possible. If diarrhea resolved completely after infliximab administration, a slow steroid taper (10 mg/week of prednisone) was carried out. In the rare patients who did not promptly respond to infliximab, further workup for infection and alternate causes of diarrhea was pursued.

The use of infliximab seems important for the prompt control of diarrhea. Previous studies suggested that one-third to two-thirds of patients with ICI-related diarrhea did not respond to high-dose IV steroids alone, or experienced relapses during a slow steroid taper, requiring an increase in the steroid dosage [25]. We believed that a prolonged steroid treatment period for diarrhea was also likely to lead to the prolonged interruption of the ICI treatment and to predispose patients to steroid-related complications. Thus, our approach of adding infliximab early in the treatment provided the opportunity to rapidly move to the more effective control of diarrhea and to decrease the time to safely resume the ICI treatment. Of note, stool testing, endoscopy, and CT scans were not routinely performed in our patients.

In our current retrospective outcome review, we found that the median time to onset of diarrhea from the start of ICI treatment was often lengthy (62.5 ± 34.5 days). In contrast, the median time from the triggering ipilimumab dose was only 18.0 ± 7.2 days. It should be noted that non-responding patients who eventually were found to have to have other defined causes of diarrhea generally exhibited a more delayed time of onset from both the start of the ICI treatment as well as from the triggering dose. Onset of diarrhea greater than 30 days after the last ICI dose was associated with a low likelihood of response to steroids or infliximab. The median time from onset of diarrhea to infliximab treatment was 7.5 ± 19.4 days in our patients. Of the 43 patients treated with infliximab for persistent diarrhea, 33 (76.7%) had rapid remission of their diarrhea, most after only a single dose. The median time to resolution of diarrhea from the first infliximab dose was 8.5 ± 16.4 days. Other studies have also demonstrated a similarly high clinical remission rate (87%) following the addition of infliximab to steroids for immune-mediated diarrhea [35].

We interpreted the response to infliximab as a strong indication that the patient had, in fact, developed bowel inflammation and colitis. If patients did not improve after the initial infliximab dose or did not completely resolve within 2 weeks after infliximab treatment, additional diagnostic testing was pursued. Evaluation included stool testing for infectious pathogens, endoscopy, and CT scans if there was the presence of abdominal pain. In these patients, other possible causes of diarrhea were usually identified. A total of 10 of our patients underwent endoscopy, and 3/10 patients were confirmed to have inflammatory colitis, which subsequently responded to vedolizumab administration. Seven patients had additional etiologies of diarrhea identified. These included ischemic colitis (*n* = 1), clostridium difficile colitis (*n* = 3), lymphocytic colitis (*n* = 1), and diarrhea from dabrafenib/trametinib treatment (*n* = 1), and unresponsive colonic inflammation due to an unknown etiology (*n* = 1).

Of our 46 original patients, 5 patients did not receive additional doses of immunotherapy, as they had already completed their planned ipilimumab monotherapy. In addition, one patient was unevaluable due to early death. Of the remaining 40 patients in our series, 30 (75%) were able to receive additional planned doses of the ICI treatment after the successful treatment of diarrhea. These 30 patients received a median of 4.5 ± 6.2 additional doses of the ICI therapy without the recurrence of diarrhea. Tumor progression was the most common cause for treatment discontinuation in the remaining 10 patients.

In other studies, it had been estimated that the continuation of ICI therapy results in a 25–34% risk of recurrent immune-mediated diarrhea [35,36]. Most of our patients received additional doses of infliximab with each subsequent treatment to prevent the recurrence of diarrhea. Continuation of infliximab during ICI therapy reintroduction seemed to prevent the risk of a diarrhea flare. This approach has also been suggested by other investigators [35,37]; however, a rigorous evaluation of the effectiveness of this approach remains to be confirmed.

Prior studies of ipilimumab plus nivolumab-treated patients reported a relatively high rate of hospitalization (or recurrent hospitalizations) compared to patients receiving PD-1 monotherapy regimens [24]. A study of 64 patients treated with ipilimumab plus nivolumab identified a 36% hospital admission rate attributed to irAE development [38]. In our patient cohort, 21.7% (10/46) of patients required hospitalizations. However, it should be noted that many of these hospitalizations were due to factors other than the severity of diarrhea. Reasons for admission included non-diarrhea-related admissions (*n* = 4), delays in insurance approvals for outpatient infliximab administration (*n* = 3), delayed initiation of treatment due to patient factors (*n* = 1), atypical presentations of colitis that did not allow expeditious treatment initiation (*n* = 1), and the development of comorbid irAEs such as hypopituitarism requiring hospitalization (*n* = 1). It should also be noted that the reported mortality rate attributed to irAEs with combination therapy is 2.3% [18]. Refractory colitis, resulting in bowel perforation and sepsis, represents important potential contributors to a fatal outcome [23]. In our current accelerated management cohort, there were no serious complications or mortality.

We were concerned that immunosuppressive treatment with steroids and infliximab might interfere with the therapeutic effectiveness of the ICI treatment. In our series, the median overall survival of our patients who developed immune-related diarrhea was 33 months, with over 40% of patients alive at 36 months follow-up. This is comparable to our previously published patient outcome following first-line ICI therapy [39]. We concluded that the use of immunosuppressive agents, such as steroids and infliximab, was not detrimental to survival. Whether the strong immune response characterized by the development of a significant irAE potentially improved clinical outcomes, especially for ipilimumab monotherapy patients, needs to be evaluated in a larger, prospective data set [40].

Our study has several important limitations. This was a retrospective review spanning a decade of patient treatment rather than a prospective study. It was based on a relatively small numbers of patients who developed diarrhea. Thus, it is mainly intended to be hypothesis-generating. A significant assumption in this study is that patients developing diarrhea were actually developing immune colitis. An initial comprehensive diagnostic evaluation was not performed. Thus, ascertainment bias is possible. Accurate ascertainment would require formal evaluation of every patient who develops diarrhea for infection and colonoscopy. This would be a logistic nightmare in a community practice and result in unnecessarily delayed care. The point of this study is to limit more extensive testing such as stool pathogen identification and endoscopy to only non-responsive patients. It is also important to note that our community practice has low rates of infections in ICI-treated patients (7%), as these patients were not receiving treatment with agents that predispose to opportunistic infection (steroids, chemotherapy, or antibiotics). Institutional patterns of infection need to be considered in adopting a diarrhea management strategy.

There was also an assumption that glucocorticosteroids followed by infliximab represented the best initial treatment for developing immune colitis versus other colitis targets (e.g., integrins, IL-23, and JAK2). The drugs utilized in our treatment approach reflect a widely used treatment sequence and are supported by multiple treatment guidelines. Infliximab proved to be effective in most of our patients, and in fact was useful as a diagnostic maneuver to identify refractory patients for further evaluation. It should be noted that three patients had infliximab-refractory immune colitis, and each of these patients responded to vedolizumab. In other series, there have been reports of more resistant and severe immune colitis treated with other measures, even including stool transplants. It is not clear whether delays in appropriate therapeutic interventions during the diagnostic evaluation resulted in more severe and resistant colitis (which we did not observe). Another assumption was that the patients seen in clinical practice are similar to those enrolled in reported clinical trials. There are likely significant differences in comorbidities due to less stringent treatment requirements in a practice setting rather than in a clinical trial setting.

## 5. Conclusions

ICIs can induce a significant spectrum of immunologic toxicities. Diarrhea and colitis are frequent complications, especially following treatment with ipilimumab-based regimens. Evaluation guidelines recommend infectious etiology workup, inflammatory biomarker testing, and endoscopy depending on the severity of the diarrhea. This testing unnecessarily delays the implementation of effective therapy. We propose that, if diarrhea responds promptly to infliximab and steroids, this is diagnostic of immune colitis. By limiting the diagnostic workup to steroid-resistant and infliximab-resistant cases of diarrhea, this is likely to result in the more effective utilization of resources, the more rapid institution of appropriate therapy, and better treatment outcomes. The implementation of this approach has not led to increased hospitalizations or resulted in treatment-related complications. The anticancer activity of ICI treatment appears to be well maintained. The continuation of planned immunotherapy is usually possible and safe following the control of diarrhea, with the continuation of infliximab treatment in conjunction with ICI treatment.

## Figures and Tables

**Table 1:** Patient demographics.

UPN	Age	Sex	Primary Site	Stage	Regimen	Comorbidities	Potential Contributory Medications	ICI Doses Prior to Colitis	Time from First ICI Dose (d)	Time from Preceding ICI Dose (d)
1	62	F		IVC	I + N	None		4	109	25
2	56	M	Axilla	IVA	I + N	HTN, hypercholesteremia		2	43	22
3	74	F	Neck	IVB	I + N	Arthritis, back pain, bronchitis/asthma, diabetes, HTN, hypercholesterolemia, GERD, chronic pain syndrome		4	76	13
4	63	M	Scalp	III—adjuvant; IVB	I	Low-back pain, SCC on left upper chest		1 *	22	22
5	75	F	Scalp	IVC	I	HTN, GERD		3	62	12
6	65	M	Mid-back	IVC	I + N	Sleep apnea, pulmonary embolism, DVT		2	25	4
7	75	M	Extr	IVA	I	HTN, hypercholesteremia, COPD, atrial fibrillation, spinal spondylosis, history of prostate cancer		4	112	49
8	85	M	Scalp	IVB	I + N	Atopic dermatitis, hypotensive heart disease, CKD, COPD, CAD, diabetes, diabetic neuropathy, duodenal ulcer, esophageal reflux, HLD, HTN, early Alzheimer’s, metabolic syndrome, peripheral neuropathy, PVD, degenerative joint disease		4	95	18
9	66	M	RCC	IV	I + N	Hypothyroidism, HTN, GERD		3	63	21
10	63	M	Extr	IV	I + N	HTN		1	22	22
11	49	M	Trunk	IVC	I	Inflammatory arthritis	D	4	107	44
12	59	M	Trunk	IIB—adjuvant	I	History of testicular cancer, HTN, hypercholesterolemia, chronic back pain, arthritis		4	128	37
13	61	M	Neck	IVB	I	Diabetes (Type II), arthritis, HTN		1	17	17
14	71	M	RCC	IV	I + N	Hypothyroidism, HTN		2	44	23
15	80	F	Vulva	IIIC (unresectable)	I	HTN, thyroid nodules, hypercholesterolemia, arthritis		1	18	18
16	51	F	Extr	IVC	I	None		1	24	24
17	64	M	Face	IIIC (adjuvant)	I	Back pain, CAD, hypercholesteremia, BPH		1	18	18
18	80	M	Trunk	IVC	I	Hypercholesterolemia, GERD, CAD, peptic ulcer disease		2	36	15
19	52	M	Trunk	IVA	I + N	Arthritis		4 **	127	40
20	70	M	Ear	IVC	I	HTN, myocardial infarction hypercholesterolemia, history of prostate cancer, history of chronic lymphocytic leukemia		2	33	13
21	76	M	Face	IVB	I	HTN, diabetes, myocardial infarction, atrial fibrillation, actinic keratoses, history of squamous cell/basal cell carcinoma, benign bowel tumor		4	116	34
22	67	M	Sinonasal	IVC	I	BPH, HTN		2	90	69
23	49	F	Vulva	IVA	I + N	Endometriosis, depression		2	46	18
24	73	F	Extr	IVC	I + N	HTN, hypothyroidism, restless leg syndrome, cyst disease in bilateral breasts		4	88	21
25	56	M	Extr	IVD	I	Unilateral kidney		6 *	47	14
26	62	F	Rectal	IVC	I + N	None		4	98	14
27	60	F	Unknown	IV	I	Hypothyroidism, hypercholesteremia, DVT, pulmonary embolism, uterine ablation, tubal ligation, scalp cyst excision, reversible posterior leukoencephalopathy		3	75	33
28	67	M	Prostate Ca	IVA	I	HTN	S	1 *	10	16
29	85	F	Extr	IVC	I + N	HTN, atrial fibrillation, cataracts, emphysema, Parkinsonism, pulmonary HTN, mitral regurgitation		3	63	2
30	62	F	Unknown	IV	I + N	None		3 *	48	6
31	61	F	Extr	IV	I + N	HTN, asthma/chronic bronchitis		3	82	9
32	25	M	Trunk		I + N	None		4	76	6
33	55	M	Extr	IIIC—unresectable	I	Myocardial infarction, HTN, history of acute renal failure	S	4	102	7
34	43	M	Extr	IVB	I	Chronic bladder inflammation, glaucoma	D, T	4	81	4
35	42	M	Unknown	IV	I + N	HTN, GERD, chronic back pain, chronic ITP		4	68	21
36	66	F	Extr	IVC	I	Morbid obesity, HTN, history of rheumatic fever, arthritis, endometrial hyperplasia		3	56	2
37	65	M	Trunk	IV	I + N	Depression, rheumatoid arthritis, low-back pain, HTN, hyperlipidemia		1	10	10
38	66	M	Scalp	IVC	I + N	History of prostate cancer		4	79	8
39	52	F	Unknown	IV	I	History of breast cancer	D, T	2	36	15
40	57	F	Extr	IV	I + N	None		N/A	N/A	N/A
41	65	F	Left groin	IVB	I	HTN, hypercholesterolemia, hypothyroidism, chronic left leg edema, small pulmonary nodules		N/A	N/A	N/A
42	61	M	Extr	IVB	I	Degenerative back problems, HTN, BPH, hypothyroidism, hypercholesteremia		N/A	N/A	N/A
43	58	F	Ear	IV	I + N	Chemical hyperthyroidism		N/A	N/A	N/A
44	60	M	Scalp	IVA	I	Asthma, depression, arthritis, BPH		N/A	N/A	N/A
45	44	M	Extr	IIB	I	Anxiety		N/A	N/A	N/A
46	64	F	Sinonasal	IVC	I	Degenerative joint disease, HTN, asthma		N/A	N/A	N/A

Sex: male (M), female (F); primary site: extremity (Extr), renal cell carcinoma (RCC), regimen: ipilimumab (I), nivolumab (N); comorbidities: hypertension (HTN), gastroesophageal reflux disease (GERD), squamous cell carcinoma (SCC), deep vein thrombosis (DVT), chronic obstructive pulmonary disease (COPD), chronic kidney disease (CKD), hyperlipidemia (HLD), peripheral vascular disease (PVD), coronary artery disease (CAD), benign prostatic hyperplasia (BPH), immune thrombocytopenic purpura (ITP); other medications: dabrafenib (D), vemurafenib (V), trametinib (T), sipuleucel-T (S). ICI doses prior to diarrhea: * retreatment patients; ** Atypical presentation: onset of abdominal pain without significant diarrhea. N/A: patients found to have non-ICI colitis.

**Table 2:** Patient outcomes.

UPN	Infliximab Doses	Time to Resolution (d)	Additional ICI Doses Post-Colitis	Hospitalization	Additional Treatment	OS (mo)	Current Status
1	1	7	0			5.1	DOD
2	1		9		Entyvio	45.4	NED
3	1	6	10			10.3	DOD
4 *	1	8	1	Diarrhea		11.8	DOD
5	3		0			2.9	DOD
6	1		3	Diarrhea; diverticulitis	Entyvio	9.5	NED
7	1	14	0 *	Metabolic encephalopathy; diarrhea		26.0	NED
8	1	2	2			5.3	Died—other
9	1	2	0			13.8	NED
10	*		2			51.4	NED
11	1	7 *	0 *			4.0	DOD
12	1	19	0 *	Infliximab *		20.5	Died—other
13	1	2	2			23.0	DOD
14	1	7	33			35.7	NED
15	1	2	0	Infliximab *		5.0	DOD
16	1	17	3			42.8	NED
17	2	14	0			3.6	DOD
18	1	3	0	Dehydration, infliximab *		5.7	DOD
19	1	14	2	Colitis (atypical presentation)		11.6	DOD
20	1	12	0			3.4	DOD
21	1	1	1	Colitis (atypical presentation)		8.4	Died—other
22	1	7 *	2			5.8	DOD
23	1	7 *	5			16.0	DOD
24	2	2	5			46.1	NED
25 *	3	14	2			33.3	DOD
26	2	14	5	Colitis (delayed management)		16.0	DOD
27	1	33	1			91.7	NED
28 *	1	16	1			57.0	DOD
29	1	2	1			15.8	DOD
30 *	*		4			11.7	DOD
31	1	9	1	Colitis, hypopituitarism		19.7	DOD
32	1	6	9			29.7	NED
33	1	7	0 *			33.0	NED
34	1	4	0 *			18.2	NED
35	1	21	6			52.8	NED
36	1	2	1			6.4	DOD
37	1		1		Entyvio	24.5	NED
38	1	8	12			45.0	NED
39	1	3	0	Progressive melanoma		1.2	DOD
40	2		0		Targeted therapy held	6.2	NED
41	2		2		Etiology unclear	7.1	DOD
42	1		0		Ischemic colitis	5.7	DOD
43	4		2		C. diff colitis	25.0	NED
44	1		1		Lymphocytic colitis	88.9	NED
45	2		0		C. diff colitis	53.5	NED
46	2		5		C. diff colitis	41.4	NED

Infliximab doses: prophylaxis *—responded to steroids alone, infliximab administered to prevent relapse. Additional ICI doses post-colitis *—patients not planned for additional doses post-colitis (received the complete 4 dose regimen). Hospitalization: infliximab *—hospitalization for treatment since insurance denied outpatient coverage for infliximab administration. Additional treatment: C. diff colitis, clostridium difficile colitis. Current status: NED, no evidence of disease; DOD, died of disease; died—other, died of non-melanoma causes.

## Data Availability

The deidentified raw data supporting the conclusions of this article will be made available by the authors upon appropriate request to the corresponding author.
